# Clinical Review, and Hospital Reports

**Published:** 1837-10-01

**Authors:** 


### Clinical Review.

### Fever in America and Ireland.

#### 1. Philadelphia Hospital.

*Differences between Dothinenteritis and the Typhus Fever, which occurred at Philadelphia in* 1836. *By W. W. Gerhard, M. D.* &c. &c.

Dr. Gerhard is an observant, a well-educated, and a talented physician. His situation at the Philadelphia Hospital has offered to him opportunities of investigating disease, of which he has amply availed himself.

The present subject is interesting, because it would appear that the typhus fever of different parts of Europe, nay, even of England and Ireland, is far from presenting uniform characters. In one place follicular inflammation and ulceration obtain; in another they do not. It is of great consequence to accumulate observations upon this point, because exclusive doctrines have been maintained even by able physicians, and false rules of treatment have been necessarily deduced from them. Let us compare the typhus of Paris with that of Philadelphia, in 1836.

"During a residence," says Dr. Gerhard, "of two or three years at Paris, I had studied with great care the pathology and treatment of the disease usually termed, in the French hospitals, typhoid fever, or typhoid affection. There is another designation for it, founded on its anatomical characters, and therefore more directly in accordance with modern medical nomenclature; it is dothinenteritis. This variety of fever, which is identical with the disease termed typhus mitior, or nervous fever, is frequent at Paris, and is almost the only fever which can be said to be endemic there. Intermittent and remittent fevers are rarely seen, except amongst those individuals who had already contracted some form of these diseases in the malarious districts of France. Some slight fevers, attended with a whitish or yellow tongue, and gastric symptoms, occasionally occur; they scarcely assume the form of a fixed disease, and usually disappear under a very simple treatment."

Dothinenteritis is the fever which has been graphically described by Louis, and which must be too familiar to our readers to demand any further notice at present. But we will accompany Dr. Gerhard to America.

"Dothinenteritis is by no means a rare disease at Philadelphia, although less common than at Paris. In the essay alluded to, I established the identity of the anatomical characters and of the symptoms of the fever, occurring at Philadelphia, with that observed at Paris. I also shewed that the patients were chiefly those who had resided but a short time at Philadelphia, and that they were taken ill on ship-board, or under some circumstances causing an abrupt change of food and habits of life. They were also young persons, but few having passed the age of twenty-five years. Both these conditions of age and change of habits are observed to be essential to the development of typhoid fever at Paris.

Having once established the complete identity of a fever which is so common at Paris, and so well described, with a similar affection, not unfrequently met with at Philadelphia, I examined the pathological phenomena of our remittent and intermittent fevers of the severe malignant character so frequently observed along the southern coast, and sometimes occurring in those malarious parts of the country which are situated within a short distance of Philadelphia. In all these fevers, the glands of Peyer, as well as the other intestinal follicles, were found perfectly healthy; the large intestine was occasionally but not constantly diseased, while the stomach, and, to a still greater degree, the liver and spleen were invariably found in a morbid condition. If the fever proved fatal in the course of the first fortnight, the liver and spleen were softened as well as enlarged; but if the disease assumed a more chronic form, the viscera were hardened as well as hypertrophied. The latter state was the first stage of these chronic lesions which are formed in the livers of patients long affected with remittents or intermittents, and which continue throughout the course of the ascites, which is so common a consequence of these diseases. I made numerous examinations of the bodies of patients who died of the same variety of malignant remittent and intermittent during the summer of 1835, and still more frequently in the epidemic of 1836, a year in which these diseases have been unusually fatal throughout the southern states. The results of these late examinations have confirmed those already obtained, and shewed that the follicles of the small intestine are free from lesions, and that the anatomical character of the disease is to be looked for in the spleen, liver, and stomach.

The bilious and yellow fevers are probably referrible to the same class as the malignant remittents; but in yellow fever the disorganization seems to be most extensive in the stomach, whence arises the black vomit, which forms a characteristic symptom of the disease. Bilious fever, or, in other words, the remittent fever attended with unusual alteration of the liver and a disordered secretion of bile, is common with us. Yellow fever is rare, and occurs in an epidemic form at such long intervals, that I have seen but few cases of it."

Dr. Gerhard turns to Great Britain, and observes that in these islands the usual type is not dothinenteritis, and that there is no constant anatomical lesion. He refers to the memoir of Dr. Lombard of Geneva, published in the Dublin Journal for September 1836, and, we may add, analyzed in our own for January 1837. He very properly adds, that full and accurate accounts of cases from British physicians are much wanted. They are so.

Dr. Gerhard goes on to state, that, in America, there have occurred several epidemics of fever, more or less similar in their nature to the British typhus. He refers to them, but the information respecting them is loose.

For a period of at least ten years there has been no epidemic of this nature at Philadelphia. In the year 1827, a large number of Irish emigrants were ill of a typhoid fever, with ulceration of the small intestines, which was probably dothinenteritis, and during several successive years there were more or less extensive epidemics of remittent and intermittent fevers, occurring in the neighbourhood of the city, but not often extending into the central parts of the town. Occasionally, sporadic cases of fever, of a comatose or typhoid character, would occur; but these cases were nearly always either some form of malignant remittent, or else they occurred during the winter months, and were complicated with pneumonia. The inflammation of the lungs then appeared as the first stage in the disease, which afterwards assumed those cerebral symptoms of stupor and feebleness which have procured for it the designation of pneumonia typhoides. At Boston, in the year 1833, there was an epidemic dothinenteritis of extreme gravity and unusually fatal.

"In the Winter of 1835-6, there was an unusual number of cases of gangrene of the lungs at the Philadelphia Hospital, and but few of decided pneumonia. Several cases of dothinenteritis occurred in the Autumn, but there were few afterwards. During the Winter, a form of fever not commonly met with at the hospitals, was observed from time to time. It was characterized by pungent burning heat of the skin, dusky aspect of the countenance, subsultus, delirium, with great stupor and prostration; but there was no diarrhœa, and but few other symptoms referrible to the alimentary canal. It was the disease which afterwards appeared as an epidemic. At first it was not well understood by us, was sometimes confounded with bronchitis or pneumonia typhoides, from the complication of pulmonary disease with the symptoms of the fever. These cases recovered under the use of a mild stimulating and supporting treatment, with one exception, in which death ensued from sloughing of the sacrum and gangrene of the lungs."

In the early part of the month of March, the admissions for the fever were more numerous, and several patients were admitted from the same neighbourhoods and same houses. This excited the attention of Dr. Gerhard, and, in company with his friend and colleague, Dr. Pennock, he instituted a strict examination of the characters of the disease. The results are the produce of the joint labours of these gentlemen; and we may content ourselves with saying that they took the utmost pains to render them as complete as possible.

The number of cases admitted with typhus, was 214. Of this number there were 120 men and 94 women. A few cases who were at the same time in the wards, and already under treatment for other diseases, are not included, although they were afterwards affected with the prevailing epidemic, but their names on the register present only the disease for which they had been admitted. The whole number of cases is, therefore, from 230 to 250. A large majority of the 214 patients were negroes or mulattoes; there were 147 people of colour and 67 whites. The disease first appeared in the former class of patients, and always prevailed more extensively amongst them than the whites who were living in the same part of the town, and exposed nearly to the same causes of disease.

The patients were obtained from various parts of the city and the neighbouring districts; but, as might be anticipated, the greatest number came from the lowest, most crowded, and most filthy quarters.

"*Classes of persons affected.*—The first patients were almost exclusively from the poorest and most intemperate class of people, chiefly day-labourers. Such was the case with most of the blacks, especially the men, who were almost without exception in the habit of drinking freely of ardent spirits. The women were without fixed occupations, or were servants out of place. As the disease extended to the different parts of the city, people of various occupations were affected; amongst them there was one respectable physician, who died of the fever. The extension of the disease to those in easy circumstances, was shewn in the practice of several eminent physicians of Philadelphia; they had not seen a case until the fever had prevailed some months at the hospital, although they afterwards met with it in their private practice."

*Mode of Propagation of the Disease.*—Its origin was unknown, as that of most epidemics. Its extension, too, and diffusion, followed the course of epidemics. But Dr. Gerhard had reason to believe it contagious. The strongest evidence was obtained in the hospital, and it is as follows:—

"Three of the principal nurses, and about a dozen assistant nurses, besides a number of patients ill with various diseases, were taken with the fever. The three principal nurses belonged, two to the wards for blacks, where there were the greatest number of fever patients, and the third to a ward for whites, where there were several cases. There was only one nurse of a ward in which many of the patients were collected, who escaped, but several of his assistants and patients were taken ill. Two of the resident physicians in attendance upon the same ward, where the patients were most numerous, were also severely ill with the fever. On the other hand, no nurse from the part of the hospital where there were but few or no typhus cases, suffered, and the number of patients taken ill in the surgical or lunatic wards was very small, not exceeding six in number. The wards in which fever patients were placed did not contain more than a third or a fourth of the population of the hospital; yet the number of cases originating in them after the first introduction of the disease, was at least four times as great as in all the other parts of the building. The alms-house and house of employment, which are separated from the hospital by a space of at least forty feet at the nearest points, furnished five or six cases, probably not more than the same number of poor in any other part of the neighbourhood would have done.

The proportion of attendants upon the sick who suffered was in exact relation to the number of fever patients in the ward; thus, in the wards for blacks (both men and women), and in the men's medical, No. 1, scarcely an assistant escaped. In the other medical wards a few were taken ill, and in the surgical and lunatic wards all the nurses escaped. The matter of the contagion, be it what it may, was generally mingled with the air, but sometimes seemed to be combined with the pungent hot sweat of the patients. In some cases the contagion was evidently direct from body to body. This was established by the evidence of a nurse and an assistant, both persons of intelligence, and, from their familiarity with the disease, quite free from fear. The nurse was shaving a man, who died in a few hours after his entrance; he inhaled his breath, which had a nauseous taste, and in an hour afterwards was taken with nausea, cephalalgia, and ringing of the ears. From that *moment* the attack of fever began, and assumed a severe character. The assistant was supporting another patient, who died soon afterwards; he felt the pungent sweat upon his skin, and was taken immediately with the symptoms of typhus. The wards in which the fever patients were placed, were large and well ventilated. We were at first disinclined to believe that the disease would prove contagious; but as soon as the fact was clearly proven, measures were taken to remove the patients not yet affected from most of these wards; and, if it had continued for a longer period, an efficient local quarantine would have been adopted. Dead bodies either did not communicate the contagion, or its influence was easily counteracted by favourable circumstances. Both Dr. Pennock and myself, and several of the resident physicians, were engaged nearly every day, during the most intense prevalence of the disease, in making long and laborious anatomical investigations, without suffering from the fever. It is very clearly proven that the typhoid fever, or dothinenteritis, is not contagious."

*Age.*—Children were very rarely attacked by the disease. After childhood, age seemed to exert but little influence.

*Sex.*—This seemed also of little moment. The numbers affected were 120 men and 94 women, about the relative proportion of ordinary patients in the hospital.

*Use of Ardent Spirits.*—A large proportion of the patients were known to be intemperate; but most day-labourers drink freely, and the number of typhus patients who were intemperate did not greatly differ from that of those affected with other acute diseases. Most of the women were not intemperate.

*Season of the Year.*—The epidemic began in March, and continued until August. There were a few scattered cases afterwards. The Summer was unusually cool, and the Spring and Winter cold. It was remarked, that as the Summer advanced, and an epidemic dysentery appeared, the fever was changed in character, and frequently offered a new symptom, that is diarrhœa, which was wanting in the earlier months.

*Colour.*—The proportion of deaths amongst the black men was much greater than amongst the whites; thus of the whites, one died in $4\frac{2}{3}$; amongst the blacks, one in $2\frac{19}{28}$. Amongst the women the reverse was true; thus, one woman died of $4\frac{3}{5}$; but only one coloured woman in $6\frac{1}{5}$ nearly. These two results would, therefore, appear contradictory, unless explained by other causes.

*Pathological Anatomy.*—Dr. Pennock and Dr. Gerhard instituted very careful pathological examinations, and neglected no opportunity of examining the dead. The results are thus recited:—

"In this large number of autopsies, amounting to about fifty, there was but in one case, and that doubtful in its diagnosis, the slightest deviation from the natural appearance of the glands of Peyer. In the case alluded to, in which there had been some diarrhœa, the agglomerated glands of the small intestine were reddened and a little thickened; but there was no ulceration and no thickening or deposit of yellow puriform matter in the submucous tissue. The disease of the glands resembled that sometimes met with in small-pox, scarlet fever or measles, rather than the specific lesion of dothinenteritis. In all other cases, the glands of Peyer were remarkably healthy in this disease, as was the surrounding mucous membrane, which was much more free from vascular injection than it is in cases of various diseases not originally affecting the small intestine.

The mesenteric glands were always found of the normal size, varying, as in health, from the size of a small grain of maize to three or four times these dimensions. With the exception of a slightly livid tint, common to them and the rest of the tissues, they offered nothing peculiar either in consistence or colour.

The spleen was of the normal aspect, in one half the cases, in the other half it was softened, but not enlarged, and in one case out of five or six, enlarged and softened.

Thus, the triple lesion of the glands of Peyer, mesenteric glands and spleen, constituting the anatomical characteristic of the dothinenteritis or typhoid fever, although sought for with the greatest care, evidently did not exist in the epidemic typhus. Indeed, it was a subject of remark, that in the typhus fever the intestines were more free from lesion than in any other disease accompanied by a febrile movement. This exemption extended to the large intestine until the summer heats began, when a few scattering cases offered some symptoms of diarrhœa, during the prevalence of an epidemic dysentery; and, where they terminated fatally, softening and other signs of inflammation of the mucous coat of the colon were observed.

The fact that the morbid changes pathognomonic of dothinenteritis, are not met with in the typhus fever, would of itself seem conclusive that the two diseases are no more identical than pneumonia and pleurisy. Although, in some respects, the two affections are analogous, and even similar, the radical difference of anatomical lesions is at least as well marked as the distinction between the symptoms. It is, indeed, singular that there should of late be a strong tendency to confound two fevers, which were regarded as entirely distinct by some of the older physicians. The prominent symptoms and difference of treatment being particularly well pointed out by Huxham."

Appended to this excellent memoir are several cases with their diurnal details, and the minutes of their dissection. These it is unnecessary for us to enter into. The memoir itself is not completed, the influence of treatment, and some other circumstances remaining for consideration. When the conclusion appears, we shall not fail to put our readers in possession of it. In the mean time, we may take the opportunity of remarking on the scientific character of Dr. Gerhard's investigations.

We shall associate with the preceding two other reports on fever, although not of Trans-Atlantic origin. They are contained in the Dublin Journal, and emanate, of course, from the Emerald Isle. The first is from Limerick.

### II. St. John's Fever Hospital.

*Report of St. John's Fever and Lock Hospitals, Limerick. By W. J. Geary, M.D., &c. &c.*

Fever prevailed extensively in Ireland in 1836, and no fewer than 3269 patients were admitted into the St. John's Hospital in that year—a number exceeding that of the average annual admissions since the regular opening of the house in 1794, by 2443, and exceeding even that of the fatal 1817-18, by 116.

The hospital itself has now "full resources" for 218 patients, and 211 have been under treatment at one time during the year.

"During the present period as well as former periods, when fever had prevailed extensively, the mortality and type of the disease do not appear to bear any regular proportion to the number attacked. If we examine the term from 1817 to 1819, we shall find that the deaths were to the recoveries as 1 in 13½—1-13½, and 1 in 18½ for each year respectively; while the numbers admitted for these periods were 1275, 2787, 3153; the disease appearing to assume a milder form as it continued to advance. The mortality during the present epidemic, which may be dated from 1835, was for that year 1 in 12½, and for 1836, 1 in 13½; the admissions for the former year being 1484, for the latter 3227."

It would appear that, since 1817, fever has been on the increase in Ireland. To what cause, asks Dr. Geary, are we to attribute this? His answer is by no means satisfactory.

"Are poverty in food and raiment, mental depression, and exposure to wet and cold, its source? If these were in themselves sufficient, we should be led to expect, that in proportion to the public exigency, we should find fever existing. It is needless to observe, that this relative condition is not in strict accordance with our experience. The due share which they manifest in the production of disease cannot be withheld from these exciting causes; but we are inclined, from very extensive opportunities of noticing the sick poor, to class them as secondary in some degree to the enervating effects of spirituous and fermented liquors, in which they but too commonly indulge to a pernicious extent. To this we may add to the total abandonment by them of any effort of cleanliness, as well as their great anxiety to keep the sick at home, to share alike their affections and wants, thus favouring the propagation of disease through contagion, under circumstances the most auspicious for its development. Are we warranted in supposing that the atmospheric constitution has undergone any alteration during the epidemic, which fits it for exercising a more effective febrile influence on the human frame? It is at least worthy of note, that the great and steady existence which fever latterly manifests, dates from that period, and should we be asked, whether the constitution of the Irish people is adapted in any peculiar degree to the reception of contagious miasm, we profess ourselves unacquainted with any information which could lead to such a conclusion."

Towards the close of 1826, the admissions became so urgent that two visiting physicians were appointed by the governors of the City Dispensary, with the view of attending the sick poor at their own houses. Dr. Geary was one of the physicians in question, and retained his situation till 1832, when he resigned. In that period, the numbers attacked with fever within the cities and liberties are susceptible of something like accurate appreciation. They are exhibited in the following table. The out-door patients are compared with the in-door patients in the period extending from the 6th January, 1827, to the 5th January, 1832.

| Year. | HOSPITAL RETURN. | | | DISPENSARY RETURN. | | | Total. |
|---|---|---|---|---|---|---|---|
| | Admitted. | Died. | Average Mortality. | Attended. | Died. | Average Mortality. | |
| 1827 | 2781 | 137 | 1 in 20 | 2800 | 80 | 1 in 35 | 5581 |
| 1828 | 854 | 37 | 1 in 23 | 960 | 22 | 1 in 39 | 1714 |
| 1829 | 506 | 23 | 1 in 22 | 640 | 18 | 1 in 35 | 1146 |
| 1830 | 806 | 34 | 1 in 23½ | 910 | 25 | 1 in 36 | 1716 |
| 1831 | 1015 | 65 | 1 in 15½ | 920 | 31 | 1 in 29 | 1935 |
| Total. | 5962 | 296 | 1 in 20 | 6130 | 176 | 1 in 34 | 12092 |

"Assuming the population at 63,310 (which is the mean of the census taken in 1821 and 1834), we shall find that the proportionate liability to fever in this district during 1827, after deducting one-sixth for county patients, and four per cent. for relapsed cases, admitted into the Fever Hospital for that year was 1 in 12½; and for the average of five years 1 in 29½: this of course is independent of the cases occurring in private practice, as well as those who may have been attacked with fever in the Liberties, and been unwilling to go into Hospital. This susceptibility to a given disease, we acknowledge to have come upon us rather by surprise, and should be somewhat incredulous on the point, if the documents from which these facts are deduced were not compiled by ourselves. Such an amount of steady and progressive disease almost tempts to the conclusion that fever has selected Ireland for its nursery, and that the Irish endemics may yet become as proverbial as yellow fever in the tropics, intermittents in the marshy districts of Holland, or malaria in Italy. We shall briefly sketch the district, which has become the theatre of such a visitation, and the extent to which poverty prevails in it. Indeed it must be obvious, that without such information, our observations must prove very defective to those who are unacquainted with our particular position."

Dr. Geary here draws a miserable picture of the poverty, crowding, and filth of the poorer classes in Limerick, who occupy the old town. The sketch that he presents is calculated to enforce the necessity for a legal provision for the poor in Ireland. The following is a tabular view of the mortality from fever:—

| Year. | Admitted. | Cured. | Died. | Average Mortality. |
|---|---|---|---|---|
| From 1794 to 1819, | 10,954 | 10,336 | 618 | 1 in 17⅙ |
| From 1820 to 1836. | 22,682 | 21,338 | 1,344 | 1 in 16 |
| Total . . . | 33,636 | 31,674 | 1,962 | 1 in 17 |

III. Navan Fever Hospital.

1. *Observations on the Use of certain Remedies in Typhous Fever, and its Complications. By Alfred Hudson, M.B. T.C.D. Physician to the Navan Fever Hospital.**

The pathology and the treatment of typhus stand the best chance of being enriched, at present, from the "seven millions." The "finest peasantry on the earth" are the victims that typhus rejoices in, and their physicians enjoy ample opportunities of observation.

We refer to the paper of Dr. Hudson, principally for the sake of ascertaining the results of his experiments with remedies exhibited in particular forms of typhus, or for particular symptoms of it. The extracts therefore must not be expected to display any necessary connexion.

A. *Treatment of Fever.* "I have been disposed to look for its indications to the condition of the viscera, and those symptoms which may daily arise, and not to the adoption of any routine system. I believe it is better to do too little than too much, and I therefore abstain from all hazardous modes of evacuation, once the disease has become established; such I conceive to be *bleeding, purging,* and *sweating.* If the type of the case be pure continued fever,

* Dublin Journal, July, 1837.

I usually content myself with ordering the diaphoretic mixture of the hospital, consisting of equal parts of camphor mixture and aqua ammoniæ acetatis, with an occasional small dose of calomel, reserving all more active measures for such complications as may arise. Of these, the most frequent is the *gastric.* In more than half the cases of synochus, the prominent symptoms were those of gastrite, and the relief afforded by leeching or cupping the epigastrium was constant and remarkable. In many cases, crisis followed immediately; in all, the patients expressed themselves relieved from prostration, weight, tightness, and similar feelings. As regards prostration in particular, my experience leads me to consider leeches, indicated by its existence in the early periods of fever, as certainly as is the necessity for wine by the same symptom in its advanced stage."

B. *Headache and Watchfulness.* "In those cases in which headach and watchfulness are complained of, I am in the habit of adding opium to the diaphoretic mixture, in the proportion of twenty-four-drops of the acetous tincture to eight ounces; and I have constantly found all the good effect described by Fordyce to follow this medicine, (3rd Dissertation, p. 236.)"

C. *Catarrh in Fever.* "Another very frequent and most serious complication of the continued fever of this country, is catarrh. As I have already stated, all our fatal cases of synochus suffered from it, and in many others it was with difficulty subdued. Antiphlogistic treatment seemed to have little power over it; when once it was established, it ran into the secreting stage, and then the patient's only chance was derived from a liberal supply of wine and nourishment, and the exhibition of the stimulating expectorant medicines: of these, the decoction of polygala, with carbonate of ammonia, was commonly given; and in very severe cases, a bolus of carbonate of ammonia, camphor, and musk, was found to be a most efficient remedy. In some instances, especially in old persons, I found warm punch of eminent service, when wine seemed to have lost power. I do not substitute it for wine in any instance; and these are the only cases in which I have seen any benefit from conjoining it; but here it produces sudden and powerful effects when wine has failed, giving force to the cough, and causing copious expectoration, restoring the warmth and natural colour of the surface, &c."

D. *Stimulation in Fever.* In the "*general* management of a case of severe continued fever, it is usually found necessary to change gradually from the expectant to a stimulating mode of treatment. I am in the habit of commencing the change (as soon as there is any appearance of the prostration of the advanced stage) by adding carbonate, or aromatic spirits of ammonia, to the diaphoretic mixture, and, as occasion requires, modifying this still farther by withdrawing the acetate of ammonia, and adding serpentaria, nitrus æther, &c., as may be indicated by the symptoms. As a general rule, also, I commence the exhibition of wine on the patient's first complaint of weakness, if the fever has advanced beyond the tenth day. I usually begin by ordering four ounces in the twenty-four hours, and increase this gradually, as may be necessary, afterwards withdrawing it in the same manner; the daily allowance seldom exceeded ten ounces: in some cases it rose to sixteen, and in a few to twenty-four ounces. The total amount of wine consumed in the two years was 267 bottles."

E. *Treatment of Gastro-enterite and Diarrhœa.* "Gastro-enterite occurred very seldom in the course of the fever. We had not in all more than twenty cases of diarrhœa, which may be attributed, I think, to several causes: 1st. The practice of leeching the epigastrium early in the disease. 2nd. Abstaining almost entirely from purgatives, and trusting to enemata for freeing the bowels; and perhaps to my seldom giving porter as a stimulant, as I found it so apt to produce purging, that I almost entirely relinquished its use.

In the instances in which diarrhœa occurred early, it yielded to the application of leeches over the cæcum, and small doses of mercury with chalk, and Dover's powder, with mucilaginous drinks. In the more advanced period, a blister to the abdomen, and a mixture of port wine and mist. cretæ succeeded very well. In some of the fatal cases, diarrhœa came on with sweating, exemplifying Baglivi's aphorism, "Si eadem tempore in acutis et gravibus morbis, duæ crisis, sudor scilicet et alvi fluxus superveniant cum pauco levamine symptomatum, fere omnes moriuntur ut sæpe vide."—*Praxeos Medicæ*, lib. i."

"F. *Chloride of Soda in Typhoid Fever.* "The general treatment and complications of typhoid fever differ materially from the preceding. As regards the former, I have found, in some instances, bad effects from even small bleedings, and should in most instances, if the typhoid type were fully ascertained, prefer relieving local congestions by blisters alone, or at most, by a very small number of leeches. As a routine practice, I think the solution of chloride of soda is to be preferred to any other, provided that it be given as soon as possible after the type of the fever is known, which in many cases means, of course, as soon as the fever has set in. I cannot say that I have seen such good effect from its use in more advanced stages, though I have prescribed it in a large number of such, and still do so; not, however, to the exclusion of any one of our tried and approved remedies. But I have noted forty-seven cases of typhoid fever in which I commenced its use as soon as the patient was admitted, or the type of the fever was evidenced by the appearance of petechiæ, &c., and in every instance with the best effect, this being, in many cases, the only medicine given; the dose was from ten to fifteen drops. In some of these cases, the effect of the chloride was evidenced by the change of colour, and diminution in number of the petechiæ, 'taches rosées,' within twenty-four hours, shewing, I think, that its action is exerted directly upon the blood, and not as a stimulant of the nervous system, as a late writer in the Dublin Journal seems (erroneously surely) to have inferred from Dr. Graves's paper on this subject.* For myself, while my limited experience leads me to place the fullest confidence in the *chemical* effects of this medicine, given *early*, I have not the least reason to attribute any stimulant powers, nor indeed any good effect whatever, to it in that stage of prostration and adynamia, which Dr. Graves has so graphically described, and in which he considers the chloride a remedy worthy of confidence."

G. *Treatment of Delirium.* "In the mildest forms, a blister applied to the neck, and small doses of opium, generally sufficed to remove it: but we had many cases in which these measures were quite insufficient; in some of them wine was found to be the best soporific. In six cases I tried Dr. Graves's mode of giving opium, viz. in combination with tartar emetic. In one, a case of furious delirium, this treatment, as well as every other, failed: in the others, and in numerous cases since the period comprised in this report, it succeeded admirably. It seems best adapted to that restless kind of delirium resembling delirium tremens, in which the patient cannot be restrained from attempting to leave his bed, and walk about the ward; when every muscle is tremulous, the eye red from want of sleep, the tongue dry, and the patient presenting that kind of spurious excitement which might induce the attendant (injudiciously, no doubt,) to order the local abstraction of blood, by leeching the temples or opening the temporal artery. I could here give reports from my note book of several cases thus treated, but that I consider it would be rendering tedious a paper already too long. In prescribing this medicine, I find it advisable to use great caution in two ways: 1st. Not to give it *after* it has produced sleep. 2nd. To follow it

---

* "Dr. Mateer's Statistics of Fever, Dub. Med. Jour. No. 28. Dr. Graves's paper, read before the British Association, August, 1835. Dublin Journal, No. 22."

up by the prompt and frequent exhibition of wine, and such nourishment or cordials as the more or less advanced stage of the disease, and debility of the patient may require; as it seems to me that there is increased risk of the patient sinking, unless timely supported after sleep thus induced."

H. *Subsultus and Hiccup.* Musk exerted most influence in controlling them.

I. *Hysteria,* frequent during convalescence, always yielded to large doses of assafœtida. In one case in which it resembled mania, a full dose of opium was of the greatest benefit.

J. *Tartar Emetic in "Indolent Pneumonia."* Dr. Hudson makes some remarks on typhoid pneumonia, but they need not detain us. He also directs attention to "an indolent form of inflammation of the lung," independent of typhus, remarkable, in his opinion, for the rapidity with which the passive congestion is resolved by tartar emetic, when mercury has failed. He cites the following as a very illustrative case.

*Case.* Dr. H. was called in consultation with another physician, in July last, to see a gentleman who had, a month before, suffered an attack of pneumonia. He had been actively treated at the outset by bleeding, blistering, and calomel, and had for some time appeared to amend gradually. For near a week, however, he had been sliding back into the same state as at first. He had cough, very considerable dyspnœa, bloody sputa, and complained of a feeling of weight and oppression in the right side. Here the signs were a general feebleness of respiration, amounting, in the lower portion, to almost perfect nullity, with dulness on percussion, and increased resonance of voice over the same portion, but *not a trace of crepitus:* respiration was puerile over the left lung. His mouth was still sore from calomel, so that its farther exhibition was out of the question; and it was agreed that he should be treated by repeated applications of leeches, and cupping-glasses to the side, and tartar emetic, in doses of a grain every third hour. After the first dose, he had the most perfect tolerance of this medicine for a week. In three days the commencement of resolution was indicated by a fine crepitus in the affected part: this gradually became more distinct for a few days, then was mixed with respiratory murmur, which by degrees became pure, until at the end of a fortnight resolution was perfected.

2. *On the Cause of Tympanitic Sound on Percussion over an Inflamed Lung. By Dr. Hudson.**

The phenomenon in question has been observed by Dr. Graves, and by Dr. Stokes, who have attributed it to air secreted in the pleura. But neither has proved the truth of the supposition by dissection.

Dr. Hudson has lately had a case, which goes to contradict the hypothesis alluded to, and, as he supposes, to establish his own. This can only be explained by a quotation.

"To those who have read Dr. Williams's remarks on percussion,† and who coincide with his views, the phenomenon will not seem incredible, nor perhaps the following *rationale* improbable.

I regard it as the necessary result of three conditions:

1st. Elasticity and tension of the parietes.

2nd. A homogeneous, solid state of the lung.

3rd. Air in the larger bronchial tubes.

The importance of the first condition may be illustrated by a drum head. That of the second is equally apparent, when we consider the effect of changes of media upon the vibrations of sound; and these, too, are necessarily associated in some cases of pneumonia, occupying the lung in its entire circumference; for till then, the tumefaction of the inflamed portion may take place at the expense of the uninflamed part; but

---

* Ibid.

† "London Med. Gazette, January."

when all becomes engaged, it must be *outwards*, and a state of tenderness of the parietes is the consequence."

The explanation must certainly be considered dubious. The following is the case.

*Case.* The patient, a young woman, was admitted into hospital on the sixth day of a pneumonia, complicated with gastro-enteritis, and which, on examination, was found to occupy the anterior and superior portion of the right lung, and a small portion of the inferior part of the left. She was apparently dying, but by repeated local bleedings, &c. &c. was relieved, and lingered for two days. On the day before her death, the right side, previously quite dull on percussion, was found to give a remarkably loud, clear sound, of a decidedly tympanitic character; much clearer, in fact, than the sound yielded by the other side, or by the stomach.

On examination of the body, it was found that:—the stomach was extensively inflamed; the lower portion of the left lung engorged; and the whole of the right lung solid, the red passing (in spots) into the grey hepatization. The pleuræ were adherent.

---

### St. George's Hospital.

### Quarterly Report of Patients treated in St. George's Hospital. By Dr. Seymour.

The following is a report of the cases treated in this hospital by Dr. Seymour, one of the physicians to the institution. In addition to his own patients, he twice admitted those of Dr. Chambers during the absence of the latter.

The cases treated during the quarter amount in number to 96. The following is a tabular view of them. It specifies the nature of the disorder—the sex of the patient—the number cured, relieved, or dead—and those who still remain under treatment. The report extends from June 19th to September 19, 1837, both inclusive.

| Name of the Disease. | No. of Patients. | Male. | Female. | Cured. | Relieved. | Died. | Under Treatment |
|---|---|---|---|---|---|---|---|
| Pneumonia .. .. .. .. .. .. | 7 | 6 | 1 | all | — | — | — |
| Rheumatic Fever .. .. .. | 5 | 3 | 2 | all | — | — | — |
| Synovial Rheumatism .. .. | 6 | 3 | 3 | 2 | 1 | — | 3 |
| Rheumatic Pericarditis—Chronic .. .. .. .. .. .. | 2 | 1 | 1 | — | — | — | — |
| Rheumatic Gout.. .. .. .. .. | 2 | 2 | — | 1 | — | — | 1 |
| Gout .. .. .. .. .. .. .. .. | 3 | 3 | — | 1 | — | — | 2 |
| Gastric Fever .. .. .. .. .. | 9 | 4 | 5 | all | — | — | — |
| Dysentery with Purpura .. .. | 2 | 2 | — | — | went away, 1 | — | 1 |
| Anasarca, from Disease of Heart .. .. .. | 4 | 4 | — | dropsy cured, 2 | — | 2 | — |
| from Disease of the Heart and Kidney | 2 | — | 2 | — | — | 1 | 1 |
| from Cold, with Syphilitic Eruption .. | 1 | 1 | — | 1 | — | — | — |
| Organic Disease of Brain .. .. | 3 | 1 | 2 | — | 2 | 1 | — |
| Paraplegia .. .. .. .. .. .. | 3 | 1 | 2 | — | 1 | — | 2 |
| Phthisis Pulmonalis .. .. .. | 4 | 3 | 1 | — | — | 4 | — |
| Bronchitis .. .. .. .. .. .. | 2 | — | 2 | 1 | — | — | 1 |
| Asthma from Dilatation of Aorta | 1 | 1 | — | — | — | — | 1 |

| NAME OF THE DISEASE. | *No. of Patients.* | *Male.* | *Female.* | *Cured.* | *Relieved.* | *Died.* | *Under Treatment* |
|---|---|---|---|---|---|---|---|
| Ascites from diseased Liver .. | 3 | 3 | — | ascites in two cases | 1 | — | — |
| Amenorrhœa with Hysteria .. | 9 | — | 9 | 6 | 3 | — | |
| Leucorrhœa.. .. .. . .. | 2 | — | 2 | — | — | — | 2 |
| Encysted Dropsy .. .. .. | 2 | — | 2 | — | by tapping, 2 | — | — |
| Large growing Tumour of the Ovarium .. .. .. .. .. | 1 | — | 1 | apparently. | — | — | — |
| Chronic Solid Tumours of the Ovarium .. .. .. .. .. | 2 | — | 2 | — | — | — | 2 |
| Tænia .. .. .. .. .. .. .. | 1 | — | 1 | — | — | — | 1 |
| Nephritis (slight) .. .. .. | 1 | — | — | 1 | — | — | — |
| Mortification of the Lower Extremities from Disease of the Heart .. .. .. .. .. | 1 | — | 1 | — | — | 1 | — |
| Epilepsy .. .. .. .. .. .. | 1 | 1 | — | — | 1 | — | — |
| Laryngitis .. .. .. .. .. .. | 1 | 1 | — | 1 | — | — | — |
| Aneurysm of the Arch of the Aorta .. .. .. .. .. .. | 1 | — | — | — | — | 1 | — |
| Icterus.. .. .. . .. .. | 1 | — | 1 | — | — | — | 1 |
| Cachectic Pains .. .. .. .. | 3 | 3 | — | 1 | — | — | 2 |
| Quotidian Ague .. .. .. .. | 1 | 1 | — | 1 | — | — | — |
| Inflammation of the Veins of the Legs .. .. .. .. .. | 1 | 1 | — | 1 | — | — | — |
| Gastrodynia .. .. .. .. | 1 | — | 1 | 1 | — | — | — |
| Bilious Vomiting .. .. .. | 1 | — | 1 | 1 | — | — | — |
| Swelling of the Inguinal Glands | 1 | 1 | — | — | — | — | 1 |
| Colica Pictonum.. .. .. .. | 1 | 1 | — | — | — | — | 1 |
| Hæmoptysis .. .. .. .. .. | 2 | 2 | — | hæmorrhage. | — | — | — |
| Pain of Head, with Discharge from the Ear .. . .. | 1 | — | 1 | — | — | — | 1 |
| Erysipelas of Feet and Head whilst nurses .. .. .. | 1 | — | 1 | — | — | 1 | — |
| Encephaloid Tubercles of Liver | 1 | — | 1 | — | — | — | 1 |
| Palpitation of Heart .. .. .. | 1 | — | 1 | — | 1 | — | — |
| Nervous Pains after Erysipelas | 1 | 1 | — | 1 | — | — | — |
| Diabetes .. .. .. .. .. .. | 1 | — | 1 | — | — | — | 1 |

*The principal acute diseases;* among these were pneumonia and acute rheumatism.

Six cases of pneumonia occurred nearly at the same time, and were attended with symptoms so remarkably similar in every respect, as to mark a peculiar form of disease. The greater number had been attacked on the same day (in June), about five days previous to admission. In all there was a nearly total inability of inspiration. The lips were blue, the pulse very quick and small, the tongue dry, and in several, delirium occurred to a great extent: in the case of Gellinan, the delirium continued unabated during several days. The blood drawn from the arm was uniformly much buffed and cupped in every case, and the treatment to subdue inflammation was successful in every case. The greater number were put upon calomel and opium (subm. hydr. gr. iij. opii, gr. j.) every four hours, with a nitre draught. After the second bleeding a blister was applied. Two of the cases occurred, however, in boys of colour (Francis and Gellinan), and experience has shewn that the natives of hot climates are easily affected with ptyalism, and often sink under it. In these cases, the use of the tartar emetic (not in the heroic doses of Italy), but in the dose of a grain every four hours, was substituted after bleeding for the calomel and opium.

In every case the solution of the disease appeared to arise from the expectoration of frothy mucus, much tinged with blood. In the majority of the cases this occurred on the third day after admission; in the worst cases on the fifth.

None of these cases proved fatal, but the convalescence of the severe ones was long.

The cases of acute rheumatism, affecting the muscles and ligaments, with swelling, the pain shifting from place to place, and attended by fever, were uniformly treated by bloodletting, ordered seldom more than once, and followed by doses of the mistura guaiaci of the Pharmacopœia, from ℥iss.—℥ij. being given every four hours, and a grain of opium at bed-time. Under this treatment the recovery was rapid in every case; in one a relapse took place.

The rheumatism principally affecting the synovial membranes, was treated with colchicum; half a drachm of the wine of the root being given twice daily, and three grains of the acetous extr., with five grains of Dover's powder at bed-time.

The cases of gastric fever were uniformly treated in the same manner, and with success.

The patients laboured under continued fever; heat of skin; red, sometimes dry, tongue; pain, often not exceeding uneasiness, in the abdomen, increased on pressing the right iliac region; thirst; want of appetite; and a greater or less degree of disturbance of the sensorium, as the individual was more or less affected by external impressions.

The stools were generally loose and ochry, sometimes watery and offensive. The treatment consisted in equal parts (gr. v.) of hydr. c̄ cretâ and Dover's powder twice or thrice daily, and castor oil sufficient to move the bowels every second day. When the pain in the abdomen was greatly increased on pressure, leeches to the caput coli, and afterwards a blister, were prescribed.

The diet consisted in diluents, and two pints of beef tea daily, very cautiously increased to white fish, without vegetables, and afterwards to meat, with a little wine.

Two very severe cases of dysentery are marked in the list, both of them with petechiæ and great prostration of strength, so as to be referred to purpura hæmorrhagica.

The first of these in which the symptoms of dysentery predominated, was treated with calomel, gr. iij. opii, gr. j. every six hours, and enough castor oil to move the bowels every morning. This patient left the hospital as soon as he felt better, but not cured.

The second, in which the petechial spots were larger, and dark-coloured, and the discharge of blood very considerable, had, in addition to calomel and opium at night, half a drachm of oil of turpentine added to the castor oil in the morning, and with the best effect. He was very rapidly relieved; affording another instance, if such were wanting, of the great effect of this medicine in intestinal hæmorrhage.

One acute case occurred of disease of the brain (Gwyne). He was admitted with symptoms of continued fever, but complained of very severe pain in the head. He had been largely bled before, and was again bled immediately after admission. On the following day he had several fits of a very peculiar character. He stretched himself forward, catching at objects at a distance; not the floccitatio which precedes death, but as if he perceived luminous objects. Several of these fits occurred, with intervals of excessive pain.

He was put on calomel and opium; the head blistered, and the blister dressed with mercurial ointment. Some temporary relief from pain appeared the consequence, but his fits returned, and in one of these he expired.

On opening the head, much fluid was found in the ventricles, but the posterior part of the right ventricle, and the thalamus on that side, was softened, of a yellow colour, and with spots of extravasated blood; and this appearance extended to the fourth ventricle and the crura cerebelli. A small quantity of semi-gelatinous fluid was contained between the membranes, over the optic nerves, and at the base of the brain. The extensive disorganization of the right thalamus, perhaps will account for the peculiar effect apparently produced on the organ of vision during the fits.

Of the irregularities of the menstrual discharge with hysteria, cases so common in the lower class of young females, it must be remarked that the irregularity consisted in almost every case in a diminished discharge, or absence of it during several months. The symptoms of distress were the well-known pain in either or both hypochondria, too severe to be inflammation, because, if proceeding from that cause, it would have been attended by great disturbance in the circulation, which was not the case; pain over the eyebrow, and sometimes globus hystericus. The shower-bath, and the preparation of assafœtida with ammonia, in severe cases, followed by steel; in less severe cases, steel, either the ammoniated tincture or Griffith's mixture, were administered with complete success; the bowels being kept open on alternate nights with 10 grains of the aloes and myrrh pill, or equal parts of pil. galbani compos. and the extr. colocynth c.

*Among the chronic cases*, the most remarkable was a case of sudden stoppage of the circulation in the lower extremities, arising in conjunction with disease of the heart.

In the last three years, three cases have occurred.

The first occurred in a middle-aged man, affected only with cough and much expectoration, slightly tinged with blood. Suddenly, when he had been a week in the hospital, while at the privy, he was attacked with intense pain in both legs, but most severe in the right. It is impossible to describe the severity of pain expressed to be felt. This was almost immediately followed by the commencing symptoms of mortification; the limbs became cold, covered with a cold sweat, and soon assumed a purple colour, reaching as high as Poupart's ligament. In this state the patient remained ten days.

On opening the body no morbid appearances presented themselves, except the singular thinness of the parietes of the heart, especially on the left side, which were also so soft as to be easily torn. A firm coagulum was formed in the common iliac on the right side, extending more than an inch, immediately before its division; and the internal membrane of the artery was discoloured, of a yellowish appearance, and softened.

The second case occurred last Spring in a middle-aged man, younger than in the last case, affected also with symptoms of catarrhal fever (influenza), with much mucous expectoration. The action of the heart was regular in this instance as in the last, but much more violent. Sudden and violent pain came on in the lower extremities, as in the former case; but motion remained equally unimpaired. A few days before death, an attack of palsy of the left side of the face took place. He survived the symptoms of mortification about five days.

On opening the body, the heart was found to be larger than natural; but the principal disease existed in the mitral valve, which was converted almost into a ring of bone, and the chordæ tendineæ greatly shortened. No disease was found in the brain; the palsy probably arising simply from the irregular distribution of blood.

The third case occurred in a middle-aged female, and was the most remarkable among the chronic diseases of the last quarter.

As this case occurred so recently, it may be related more in detail.

Sarah Ridge, æt. 39, servant, admitted July 13.

Two days ago, was seized with violent pain in the head, and sickness, intense pain in the legs supervening; has complained of palpitation of the heart and dyspnœa occasionally for the last 15 months; at present the extremities are cold, and manifestly suffering from want of circulation; there is continued violent action of the heart; pulse scarcely perceptible at the wrist; bowels not open from medicine ordered on admission; has been bled without relief.

Hydr. subm. gr. iij.

Opii, gr. ¼, 4tis horis.

Ol. ricini ℥ss.

Fotus papaveris cruribus assedue admovend. Enema domesticum statim.

V. S. ad ℥x.

Balneum vaporis post vænesectionem.
Opii gr. i. 4tis horis.

15th. Blood drawn not buffed or cupped; pulse very weak, almost imperceptible; pains have ceased in the limbs; has taken a draught with acetatis morphiæ gr. i., from which she appeared greatly relieved.

Mortification proceeding rapidly in the left leg.

Rep. H. c̄ morphiæ acet.

16th. Pulse rather stronger; mortification proceeding in both legs; no pain.

Died at a quarter past 10 o'clock.

Post mortem examination on the 18th.

The cuticle over the instep and ankle had been raised into vesicles; the skin beneath being very dark-coloured, and the integuments of the legs generally (especially of the left) had the stained and mottled-brown appearance of incipient sphacelation. The heart was very large and flabby, rather more than the usual quantity of fluid in the pericardium. The mitral valve was thickened, and its chordæ tendineæ unusually short, and the curved columnæ very much increased in bulk, but so contracted as by their further contraction, under action, materially to have interfered with the function of the valve. There was a considerable semi-cartilaginous deposit of very irregular form beneath the serous lining of the left ventricle, near its upper part or base, at the junction of ventricle and auricle. The lungs, with the exception of slight adhesions, were unusually healthy in structure.

The artery from the common iliac to the popliteal space in the left leg was much stained with the colouring matter of the blood, and appeared to be much congested, and its vasa vasorum were extremely injected. The veins were quite healthy—the right leg was not examined, as the mortification had advanced in a less degree in that extremity.

The above-mentioned cases may be considered rare, as three only have occurred in the extensive observation of a physician to several institutions.

They are not to be considered as arising from palsy, inasmuch as in two out of three cases the brain was entirely unaffected on examination; in the third it was not examined, for there was no delirium, the senses were entire, the power of motion in the affected limbs was not altered, and the sensibility most materially encreased.

Neither can they be compared with cases of mortification of the lower extremities, with vesications, in anasarca from disease of the heart. In such cases, the disease comes on after protracted illness, either in the last days of life or after erysipelas of the lower extremities. In the examples mentioned there was no dropsy; the attack of pain was *instantaneous,* as if the limbs were deprived of the due supply of blood suddenly and decisively.

A remarkable case of tape-worm, (tinea lata) was among the chronic cases. The patient passes at stool every day joints of this animal, usually more than fifty, occasionally five or six are joined together, but more commonly they are separately. During the last two months she must have passed several thousand joints of about nearly half an inch in length. Her general health is good, but she complains of a dragging pain in the left side and round the umbilicus; but she has not become thin or pale.

The remedy employed, and which has never failed to bring away these broken pieces of the worm, is the root of the male fern, (aspidium filix mas) half a drachm of the powder being given thrice daily, and every other day a powder of calomel, scammony and rhubarb, or half an ounce of castor-oil. Oil of turpentine has not been so effectual, and produced much sickness.

Another chronic case, that of Wm. Cannon, excited much attention. He was a man of about 50 years of age, with enormous fullness of the face and throat, suffusion of the eyes, like one threatened with apoplexy. There was much disturbance about the hearts' action, as if enlarged and dilated—no pulsation in the right wrist or arm; he had undergone the operation for popliteal aneurysm in the right leg about three years ago, and subsequently the limb was amputated.

The entire stoppage of the circulation in the left arm, together with the fact of aneurysm having occurred previously, and the disturbance in the hearts' action, suggested aneurysm of the innominata.

The patient was bled repeatedly, always with relief, and the first bleedings shewed a very strong buffy coat. He was put as far as possible in a hospital on dry diet, a practice, liable always, however, to be defeated by the patient receiving drink from his fellow patients. He suffered greatly from difficulty of swallowing, and the last two days from cough. He expired suddenly on the 17th of September, having been one month in the hospital.

*Sectio Cadaveris.* The right cavity of the chest was found filled with coagulated blood, which had escaped by the bursting of an aneurysm, which occupied the origin of the aorta immediately above the semilunar valves, but did not implicate the three great vessels.

The cyst adhered to the superior lobe of the right lung, and had, as it were, buried itself in its substance, to which it was intimately united. It contained some loose and partially adherent, but no distinct laminated coagulum. It had given way on the outer side, and the blood had been poured through a small part of the substance of the upper lobe of the lung, and through a rent in the pleura stretching across the interlobular fissure, into the pleural cavity.

The coats of the aorta were much diseased. The middle coat was the seat of abundant atheromatous deposits, and the internal coat was to all appearance destroyed in the aneurysmal pouch.

At the orifice of the right subclavian artery the inner coat had been dissected, as it were, from the middle, and formed a very decided valve, which must necessarily have been thrown up at each systole of the heart. This, of course, prevented the free ingress of blood into the artery, and accounted for the absence of a pulse at the right wrist.

The heart itself was large, and its cavities dilated. But its walls could not be said to be unnaturally thick.

